# Surgical treatment for lumbar tuberculosis by posterior transforaminal lumbar debridement, interbody fusion, and instrumentation in the aged

**DOI:** 10.1186/s40064-016-2243-0

**Published:** 2016-05-13

**Authors:** Bo Yu, Yu He

**Affiliations:** Department of Critical Care Medicine, Second Xiangya Hospital, Central South University, 139 Renmin Road, Changsha, 410011 Hunan People’s Republic of China; Department of Radiology, Second Xiangya Hospital, Central South University, 139 Renmin Road, Changsha, 410011 Hunan People’s Republic of China

## Abstract

**Object:**

To evaluate the clinical efficacy and feasibility of single-stage posterior debridement, interbody fusion and posterior instrumentation for the treatment of lumbar tuberculosis in the aged and to discuss the surgical strategies of this intervention.

**Methods:**

From January 2006 to January 2012, 28 elderly patients who suffered from lumbar tuberculosis underwent one-stage posterior debridement, interbody fusion and posterior instrumentation. Radiographic data which included correction of local kyphosis, loss of correction and bone fusion were carefully collected pre and postoperatively to evaluate the efficacy of surgery. Perioperative and postoperative complications were also registered. All patients were asked to fill out Oswestry Disability Index questionnaire before the surgery and at the last follow-up.

**Results:**

All patients (12M/16F) were followed for at least 24 months. The average kyphotic angle decreased to 11.3° ± 7.0° postoperatively from 26.4° ± 5.7° preoperatively. Meanwhile, average loss of 2.0° ± 1.5° was observed at last visit. Bone fusion occurred at 4–6 months. Neither mortalities nor any neurological complications were found in the series. 16 cases who suffered neurologic insults before surgery, the majority of patients recovered after surgery. The mean Oswestry Disability Index was significantly improved from 28.6 ± 4.9 before surgery to 10.4 ± 3.8 at last visit.

**Conclusions:**

The outcomes of follow-up showed that single-stage posterior debridement, interbody fusion and instrumentation is an effective method for the treatment of lumbar tuberculosis in the aged.

## Background

Spinal tuberculosis (STB) is one of the oldest diseases known to mankind and has been found in Egyptian mummies dating back to 3400 BC (Taylor et al. 2007). The disease is well known as Pott’s spine. It is a frequently encountered extra-pulmonary form of the disease. It accounts for approximately half of all cases of musculoskeletal TB (Turgut 2001). It is characterized by formation of cold abscess, destruction of the intervertebral disc and the adjacent vertebral bodies, collapse of the spinal elements and anterior wedging leading to kyphosis (Huang et al. 2014).

China is one of the world’s two TB high burden countries, and ranked second in the incidence of TB (Wells et al. 2010). It has been an important public health issue, accompanying with serious medical, social and financial impacts. With an increase in STB incidence and the rising proportion of aging population in China, STB in elderly patients is on the uptrend (Vetillard et al. 2012).

The aim of surgery for STB is to debride the focus thoroughly, improve neurological status by removal of compressive elements and restore the spinal stability helping in healing and bone fusion. Reviewing previous literature, various surgical approaches have been recommended for treating lumbar TB including anterior approach considered as a gold standard (Li et al. 2014; Wang et al. 2013; Yang et al. 2013), one- or two-stage combined anterior–posterior approach (Wang et al. 2012a, 2013; Zhang et al. 2012a), or posterior approach alone (Wang et al. 2012a, b; Liu et al. 2014; Sahoo et al. 2012; Zhang et al. 2013). The aged STB patients as a special population usually coexists with spinal degeneration disorders, osteoporosis and other comorbidities, which made the treatments more difficult. Moreover, there is a paucity of research about surgical strategies of lumbar STB treatment in the old patients. Therefore, in this study, we present the old patients with lumbar TB treated by single-stage posterior debridement, interbody fusion and instrumentation.

## Methods

### Basic information

Written informed consent was acquired from each of the patients to authorize treatment, radiological data, and photographic documentation. The study protocol was approved by Second Xiangya Hospital Ethics Committee. 28 aged patients averaged 72.3 ± 8.6 years old, with lumbar tuberculosis who had been treated in our hospital from September 2006 to September 2011 were enrolled in this study. The cohort comprised 12 males and 16 females, with a minimum 2-year follow up (Table 1). The diagnosis of lumbar TB was based on clinical symptoms, radiologic presentations and pathological examinations (Currie et al. 2011). Plain radiology, computed tomography and MRI of the spine were performed on all patients admitted with suspected spinal tuberculosis. Neurological assessments were done using the Frankel scoring system (Davis et al. 1993). Kyphotic angel was measured on the plain radiology, by drawing two lines–one was along the top surface of the immediate upper normal vertebral body, and the other was away from the diseased segment. The indications for posterior surgery included the progressive neurological deficit, spinal instability, kyphotic deformity (kyphotic angel <60°), refractory disease, epidural abscess compressing the dural sac, posterior disease and the lesion confined to less than two adjacent segments. The lesion involved more than 2 adjacent segments, multilevel non-contiguous involvement, severe tubercular kyphosis or had large paraspinal abscess were excluded in this study. All patients were prescribed anti-TB drugs (rifampicin: 10 mg/kg, isoniazid: 5 mg/kg, ethambutol: 15 mg/kg, pyrazinamide: 25 mg/kg) 2 weeks before the operation.

**Table 1:** Demographic data

Parameters	No.
Gender (M/F)	12/16
Average age (years)	72.3 ± 8.6
Number of vertebrae affected	
One	9
Two	19
Preop. Frankel grade	
Grade A	0
Grade B	0
Grade C	4
Grade D	12
Grade E	12
Distribution of lumbar tuberculosis	
T12 + L1	1
L1	2
L1 + L2	3
L2	1
L2 + L3	5
L3	2
L3 + L4	5
L4	4
L4 + L5	5
Preo. Kyphosis angle	30.9 ± 10.5

### Surgical procedure

After administration of general anesthesia with somatosensory-evoked potential monitoring, surgery starts by identification and exposure of the affected level. An adequate number of transpedicular screws (Moss Miami, DePuy Spine, Raynham, MA) are inserted proximally and distally based on the bone quality and the number of involved vertebras, trying to reserve valuable motion segments without jeopardizing fixation adequacy. Then a temporary stabilizing rod is fixed unilaterally. A laminotomy and facetectomy are performed at the affected level. Expose the affected disc and destructed vertebrae body and curette them with various curettes and shavers until bleeding bony surfaces are reached. Meanwhile, spinal core decompression is performed or a psoas abscess usually drains in this stage. Appropriately sized autogenous tricortical iliac crest grafts together with sufficient cancellous bone chips are implanted in the bone defect. Then autogenous bone or allograft (Aorui Biological Material, ShanXi Province, China) was selected for posterior fusion at the addressed segments. In the end, drainage and incision sutures are performed. The debrided specimens are histopathologically examined.

### Post-operative management and follow-up

Typically, the drain was removed when drainage flow was less than 50 mL/day. Patients were recommended to walk around with the effective support of a plastic orthosis, after remaining supine for 1–2 weeks postoperatively. Anti-TB chemotherapy with the four drugs (isoniazid/rifampicin/ethambutol/pyrazinamide) was performed for at least 4 months, then followed by rifampicin/INH/pyrazinamide for a further 9 months, until regression of symptoms, and resolution of laboratory and radiological abnormalities.

The following indexes including radiologic examinations, hematologic parameters and neurological function, were recorded pre-, postoperatively, and at the last follow-up. The bone fusion was evaluated according to the following criterion (Table 2). A and B were regarded as successful fusions, and C and D were regarded as failure of fusion (Lee et al. 1995). The Oswestry disability index questionnaire (ODI) was administered to all patients for clinical outcome assessment.

**Table 2:** Evaluation criteria of bone fusion

A	Definitive fusion: definitive bony trabecular briding across the graft interface, no motion on flexion–extension X-ray films, and no gap at the interface
B	Probable fusion: no definitive bony trabecular crossing, but no detectable motion and no identifiable gap at the interface
C	Possible pseudarthrosis: no bony trabecular crossing, no motion, but identifiable gap at the interface
D	Definite pseudarthrosis: no traversing trabecular bone, definitive gap, and motion greater than 3°

### Statistical analysis

The statistical analysis was performed using GraphPad Prism software Version 6.0 (San Diego, CA, USA). Results were presented as the mean ± SD. Statistical analysis was performed with Student’s paired t test and repeated measure ANOVA. Differences were considered statistically significant if the *p* < 0.05.

## Results

### Operative results

All patients were treated by posterior debridement, transforaminal interbody fusion with instrumentation. The length of surgery was 262.9 ± 32.3 min. Blood loss during surgery was 433 ± 107 mL. No wound infection and sinus formation occurred. None died of perioperative complications. No complications related to instrumentation occurred in all cases. The overall mean follow-up was 35.1 ± 7.7 months.

**Fig. 1:**
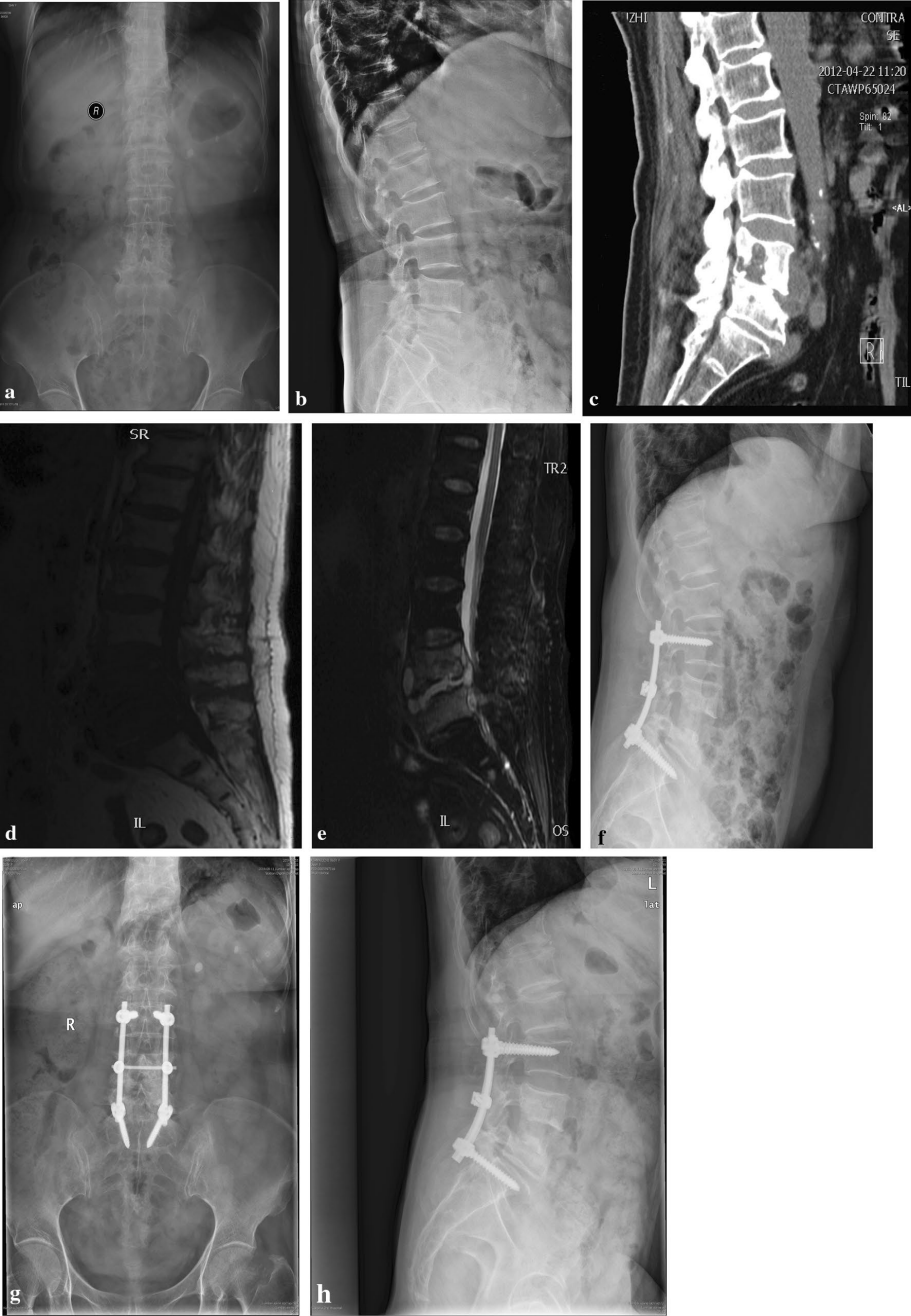
Images in a 64-year-old woman with L4 and L5 tuberculous spondylitis. **a**, **b** Preoperative X-ray films demonstrate collapse of the L4 and L5 vertebras with narrow intervertebral space. **c**–**e** Preoperative sagittal CT and T1 and T2 W MRIs demonstrate bone destruction and posterior epidural abscess at L4/5. **f** Immediate postoperative X-film showed that the patient received posterior transforaminal lumbar debridement, interbody fusion and instrumentation at the lesion. **g**, **h** X-ray films reveal bone fusion and internal fixation in good position at 2-year follow-up

### Radiographic and hematologic results

The solid bone fusion was obtained in all patients at the final follow-up (Fig. 1). There were 16 patients at level A and 12 patients at level B. The serum level of ESR returned from 52.9 ± 26.4 mm/h preoperatively to normal with 3 months. The kyphotic angle improved from 26.4° ± 5.7° preoperatively to 11.3° ± 7.0°, with a mean correction loss of 2.0° ± 1.5° at the final visit (F = 49.27, *p* < 0.0001, Table 3).

**Table 3:** Radiographic results

	Pre-operation	Post-operation	Final follow up	Correction*	Loss of correction
Kyphosis angle (°)	30.9 ± 10.5	17.2 ± 3.5	18.8 ± 1.3	13.7 ± 3.8	1.6 ± 1.1

* Statistically significant difference comparing preoperative and postoperative values (t = 8.811, p < 0.001)

### Neurological status

No neurological deterioration was found in the series postoperatively. 16 cases suffered neurologic insults before surgery, and 13 cases recovered after surgery. At latest follow-up, most demonstrated normal neurological functions, Frankel C and D were observed in one and two cases respectively (Table 4).

**Table 4:** Neurologic recovery according to Frankel scoring system

Preoperation		Final follow-up		
		C	D	E
C	4	1	2	1
D	12			12
E	12			12

### Clinical outcome

**Fig. 2:**
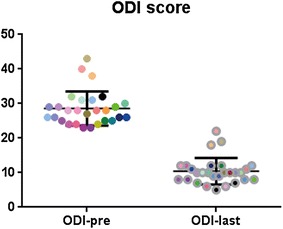
Scatter plot shows the ODI scores of every patient preoperatively and at final follow-up, with significant difference (t = 19.19, p < 0.0001)

The mean ODI improved from 28.6 ± 4.9 preoperatively to 10.4 ± 3.8 at the latest visit (t = 19.19, p < 0.0001, Fig. 2).

## Discussion

Treatment for spinal tuberculosis is still controversial. Anti-TB chemotherapy is the mainstay in controlling and treating the disease. Moreover, surgical management is an effective choice for the severe spinal tuberculosis, which can relieve pain symptoms, improve neurological function and reconstruct the spine stability. Various methods of surgical treatment in patients with lumbar spinal TB have been reported. The involvement always affects the anterior column of the spine, namely, the disc and the adjacent vertebral bodies; therefore, anterior approach was often recommended to evacuate an abscess, excise the necrotic tissues, decompress the neural tissues, and to insert a bone graft to correct kyphosis, achieve solid fusion and minimize disease recurrence (El-Sharkawi and Said 2012). However, anterior exposure of the abdominal blood vessels and ureter presents a significant challenge to the spine surgeon, especially when vertebras destruction by infection leads to severe kyphosis. Some researchers advocated anterior debridement and posterior instrumentation in one or two stages and emphasized its advantages as reaching the lesion directly and decompressing the spinal cord effectively (Hirakawa et al. 2010). Nevertheless, it is more invasive than single stage procedure. Zaveri (Zaveri and Mehta 2009) firstly reported on 15 cases of lumbar tuberculosis by transforaminal lumbar interbody fusion and posterior fixation (TLIF procedure) and received good clinical outcomes. It is far away from the ureteral and abdominal artery and characterized as a simple approach. Moreover, it also provides adequate exposure of the anterior part of the spinal canal and affords efficient decompression rendering good clinical results. Subsequently, this procedure is widely applied for treating thoracic and lumbar tuberculosis (Huang et al. 2014; Zhang et al. 2012b, 2013). In this study, we applied the similar procedure for lumbar tuberculosis in aged patients and also achieved good clinical outcomes at the final follow-up.

Lumbar tuberculosis usually causes severe kyphotic deformity which was directly correlated with the low back pain (Glassman et al. 2005; Roussouly and Nnadi 2010). As we know, transpedicular screw fixation could provide sufficient stabilization and obviate the progress of late angular deformity. Spinal biomechanical stabilization provides appropriate conditions to treat tuberculosis, assists to suppress the infection and affords a relatively stable internal environment to decrease tuberculosis recurrence. Moreover, it has been demonstrated experimentally to improve neurological recovery (Vaccaro et al. 2006). In this series, the majority of patients achieved significant improvement in daily activities (Fig. 2) and neurologic deficits at the final follow-up (Table 4).

From our experience, the treatment of spinal TB in the aged should focus on the following points. First, in elderly patients with spinal TB, spinal degeneration existed and the relatively narrow spinal canal can easily result in nerve damage and even paralysis in the early stage. Second, old patients usually have comorbidites, so a thorough examination is very important before the operation, which guarantees the surgery smooth. Third, osteoporosis is a constant feature of the disease in old patients (El-Sharkawi and Said 2012). It may result in implant loosening and back pain. If unaddressed, it may compromise the stability of spine. Therefore, to reduce the risk of fixation failure and poor results, posterior long-segmental instrumentation is generally necessary. In addition, the patients with severe osteoporosis have been given calcitonin for treatment of osteoporosis for 1–3 months after surgery.

The posterior debridement surgery has its limitations. It is difficult to complete anterior–lateral debridement, whiles the operation never overemphasize radical debridement as the tuberculous lesion may heal in spontaneous fusion under the chemotherapy. Besides, it is hard to remove large and sticky abscess. Similarly, when the aged has poor bone fusion, tuberculosis recurrence, anterior column collapse or posterior process deformity aggravation, anterior debridement plus ventral stabilization of spine is necessary.

## Conclusion

On the basis of the results of this study, it is concluded that single-stage posterior debridement, interbody fusion and posterior instrumentation can be an effective treatment method for the lumbar TB. This method can relieve pain symptoms, improve neurological function and reconstruct the spine stability.
